# Lung perfusion disturbances in nonhospitalized post‐COVID with dyspnea—A magnetic resonance imaging feasibility study

**DOI:** 10.1111/joim.13558

**Published:** 2022-08-26

**Authors:** Jimmy Z. Yu, Tobias Granberg, Roya Shams, Sven Petersson, Magnus Sköld, Sven Nyrén, Johan Lundberg

**Affiliations:** ^1^ Department of Radiology Solna Karolinska University Hospital Stockholm Sweden; ^2^ Department of Molecular Medicine and Surgery Karolinska Institutet Stockholm Sweden; ^3^ Department of Neuroradiology Karolinska University Hospital Stockholm Sweden; ^4^ Department of Clinical Neuroscience Karolinska Institutet Stockholm Sweden; ^5^ Department of Medical Radiation Physics and Nuclear Medicine Karolinska University Hospital Stockholm Sweden; ^6^ Department of Respiratory Medicine and Allergy Karolinska University Hospital Stockholm Sweden; ^7^ Department of Medicine Solna Karolinska Institutet Stockholm Sweden

**Keywords:** biomarkers, COVID‐19, dyspnea, magnetic resonance imaging, perfusion

## Abstract

**Background:**

Dyspnea is common after COVID‐19. Though the underlying mechanisms are largely unknown, lung perfusion abnormalities could contribute to lingering dyspnea.

**Objectives:**

To detect pulmonary perfusion disturbances in nonhospitalized individuals with the post‐COVID condition and persistent dyspnea 4–13 months after the disease onset.

**Methods:**

Individuals with dyspnea and matched healthy controls were recruited for dynamic contrast‐enhanced magnetic resonance imaging (DCE‐MRI), a 6‐min walk test, and an assessment of dyspnea. The DCE‐MRI was quantified using two parametric values: mean time to peak (TTP) and TTP ratio, reflecting the total lung perfusion resistance and the fraction of lung with delayed perfusion, respectively.

**Results:**

Twenty‐eight persons with persistent dyspnea (mean age 46.5 ± 8.0 years, 75% women) and 22 controls (mean age 44.1 ± 10.8 years, 73% women) were included. There was no systematic sex difference in dyspnea. The post‐COVID group had no focal perfusion deficits but had higher mean pulmonary TTP (0.43 ± 0.04 vs. 0.41 ± 0.03, *p* = 0.011) and TTP ratio (0.096 ± 0.052 vs. 0.068 ± 0.027, *p* = 0.032). Post‐COVID males had the highest mean TTP of 0.47 ± 0.02 and TTP ratio of 0.160 ± 0.039 compared to male controls and post‐COVID females (*p* = 0.001 and *p* < 0.001, respectively). Correlations between dyspnea and perfusion parameters were demonstrated in males (*r* = 0.83, *p* < 0.001 for mean TTP; *r* = 0.76, *p* = 0.003 for TTP ratio), but not in females.

**Conclusions:**

DCE‐MRI demonstrated late contrast bolus arrival in males with post‐COVID dyspnea, suggestive of primary vascular lesions or secondary effects of hypoxic vasoconstriction. Since this effect was not regularly observed in female patients, our findings suggest sex differences in the mechanisms underlying post‐COVID dyspnea, which warrants further investigation in dedicated trials.

## Graphical abstract

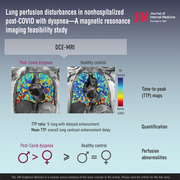

## Introduction

Post‐COVID‐19 condition, also known as “long‐COVID” or post‐acute COVID‐19 syndrome, is prevalent and can persist for months after the acute illness [1]. One prominent symptom is dyspnea, found in 24.5% of hospitalized patients and 39.9% of nonhospitalized patients 60 days after onset of infection [2]. In hospitalized patients, potential explanations include fibrotic‐like changes visualized by computed tomography (CT), reduced spirometry volumes, and diffusion capacity [3, 4, 5, 6]. However, a sizeable group of nonhospitalized patients also have a high incidence of dyspnea, with scarce objective findings on CT and lung function tests, but the nonhospitalized patient group is not well studied [7]. In these patients, the pathophysiological mechanisms are less clear.

During the acute SARS‐CoV‐2 infection, the lungs are the primary site of infection, developing into, in the worst case, an acute respiratory distress syndrome (ARDS) pattern [8, 9, 10]. Pulmonary involvement leads to local tissue disruption, including microvascular damage. Clinical reports support similar findings regarding an association between pulmonary hypertension and more severe disease [11]. Radiological methods including contrast‐enhanced CT, dual‐energy CT (DECT), and single‐photon emission CT (SPECT) combined with CT indicate disturbances in pulmonary blood distribution [12, 13, 14, 15, 16, 17]. The combined impression of unequal blood distribution in single time point scans (DECT) or composite tracer distribution (SPECT) has further support from findings in a case report of an intensive care unit (ICU) patient using dynamic contrast‐enhanced magnetic resonance imaging (DCE‐MRI) [18]. It has been speculated that lung perfusion disturbances could partly explain the clinical deterioration in the acute phase [19]. The importance of examining lung perfusion in post‐COVID patients has been stressed in one exploratory SPECT‐CT study [20]. Indeed, early SPECT‐CT studies in nonhospitalized post‐COVID patients have suggested residual perfusion disturbances [21].

Blood flow in the lungs can be studied using pulmonary angiography, DECT, SPECT‐CT, and DCE‐MRI. The essential advantage that sets DCE‐MRI apart from the former methods is the ability to render both spatial and temporal information with a reasonable resolution, enabling the detection of subtle perfusion impairments and possibly shunts. DCE‐MRI is a clinically applied method for brain perfusion imaging and has also been successfully applied in lung diseases such as chronic obstructive pulmonary disease, cystic fibrosis, pulmonary embolism, pulmonary artery (PA) stenosis, and pulmonary vasculitis [22, 23]. In addition, the ability to process DCE‐MRI parametric maps into a few summarizing numeric values facilitates quantitative comparisons between different individuals and time points. The lack of ionizing radiation is advantageous too, especially for repeated examinations of young persons.

Clinical DCE‐MRI typically relies on visual assessment of focal areas of altered blood flow. [24]. However, when the entire organ has pathological perfusion without focal disturbances, both visual assessments and ratios can fail to deliver absolute and comparable data [25]. To address this, we have previously constructed a methodology that normalizes the local contrast bolus peak time against the mean global passage of the bolus [18]. Succinctly, every voxel is normalized in the time domain between 0 (the peak in the pulmonary trunk) and 1 (the peak in the aorta) and summarized in two parameters: mean time to peak (TTP‐mean) and time‐to‐peak ratio (TTP‐ratio). TTP‐mean captures both heterogeneities in the time domain and possible relative shifts against the average global transfer time, indicating changes in vascular resistance. TTP‐ratio captures voxels with significantly delayed arrival compared to the global average bolus transfer, capturing potential shunting away from these areas or collateralized flow.

We hypothesized that lung perfusion disturbances might exist in the post‐COVID condition and contribute to dyspnea. We, therefore, prospectively applied DCE‐MRI to investigate whether pulmonary perfusion disturbance exists in nonhospitalized post‐COVID individuals and whether it might be associated with dyspnea.

## Methods

### Ethical considerations

The Regional Ethics Review Board in Stockholm and the Swedish Ethical Review Authority approved this prospective cross‐sectional study performed between October 2020 and May 2021 (original approval number 2018/2416‐31 with amendments 2020‐00047, 2020–02535, and 2021‐00815). Written informed consent was obtained from all participants.

### Participant selection and enrollment

Inclusion criteria for the post‐COVID condition group were a history of past COVID‐19 infection, verified by real‐time polymerase chain reaction (RT‐PCR) and persistent dyspnea at enrollment. Participants were recruited through a patient network in Sweden and were kindly asked to recruit an age‐ and sex‐matched healthy control, if possible. Controls were included if they had a negative antibody test within 3 weeks of the imaging session and absence of COVID‐suspect symptoms since the pandemic started.

Exclusion criteria for both groups were (i) a history of smoking for more than 5 years and (ii) any cardiovascular or pulmonary conditions requiring medical follow‐up or treatment. In addition, all participants were asked to fill out an MRI safety checklist, and any contraindications meant exclusion from the study.

One person with idiopathic pulmonary fibrosis, diagnosed by the Respiratory Medicine Clinic at Karolinska University Hospital, was additionally included as a positive control. He was in his 80s (more than 4 standard deviations [SD] older than the post‐COVID group), with a body mass index (BMI) of 23.8 (within 1 SD). His disease severity was assessed as “mild to moderate” [26, 27] based on a forced vital capacity of 92% and predicted and diffusion capacity of 63%, and did not require long‐term oxygen treatment. Thoracic CT showed subpleural reticular changes with traction bronchiectasis and no honeycombing. The total volume of morphologic changes was visually assessed to be 10%–20% of the total lung volume.

### Clinical data acquisition

All clinical data were collected directly in conjunction with the MRI scanning session. Essential patient characteristics were recorded: age, sex, body height, body weight from which the BMI was calculated, date of disease onset, and confirmed RT‐PCR test result.

Dyspnea severity was quantified through two validated self‐reported symptom scales: the modified Medical Research Council dyspnea scale (mMRC) and Chronic Obstructive Pulmonary Disease Assessment Test (CAT) [28, 29]. The CAT scale includes several questions regarding different symptoms related to COPD. Only one question, the one related to exertional dyspnea, was used in the analysis. In both scales, a higher number means more dyspnea. Subjective exertional impairment was quantified through the validated self‐reported Frändin–Grimby [30]. A lower number means less daily physical activity.

Objective exertional impairment was quantified through a 6‐min walk test (6MWT), performed according to the American Thoracic Society 2002 guidelines by J. Y. (pulmonologist and fifth‐year radiology resident) [31]. We also related the absolute walking distance to expected values based on normative data [32] to account for differences in age, body height, and body weight, which might affect the absolute walking distance.

### MRI image acquisition

Pulmonary imaging was performed by J. Y. and R. S. (MRI technologist) under the supervision of T. G. (radiologist) on a Siemens MAGNETOM Skyra 3 Tesla MRI scanner (Siemens Healthineers, Erlangen, Germany). The imaging protocol was designed by J. Y., T. G., S. P. (MRI physicist), and J. L. (radiologist), including three morphological sequences and one perfusion sequence. Prior to entering the scanner, the patient was given breath‐hold training. The entire imaging session lasted 15–20 min.

Three morphological nonenhanced sequences, each performed during inspiration breath‐hold for 16 s, were used to identify lung opacifications: a coronal 2D T2‐weighted half‐Fourier single‐shot spin‐echo (“HASTE,” field of view 400 × 400 mm, voxel size 2.1 × 2.1 × 5 mm, echo/repetition times at 23/400 ms, 36 slices), a transverse 2D T1‐weighted spoiled gradient echo (“VIBE,” field of view 262 × 400 mm, voxel size 1.0 × 1.0 × 4 mm, flip angle 5°, echo/repetition times at 1.9/4 ms, 72 slices), and a coronal 3D ultrashort echo‐time spiral VIBE (field of view 600 × 600 mm, voxel size 2.3 × 2.3 × 2.3 mm, flip angle 5°, echo/repetition times at 0.05/2.62 ms, 104 slices).

**Fig. 1:**
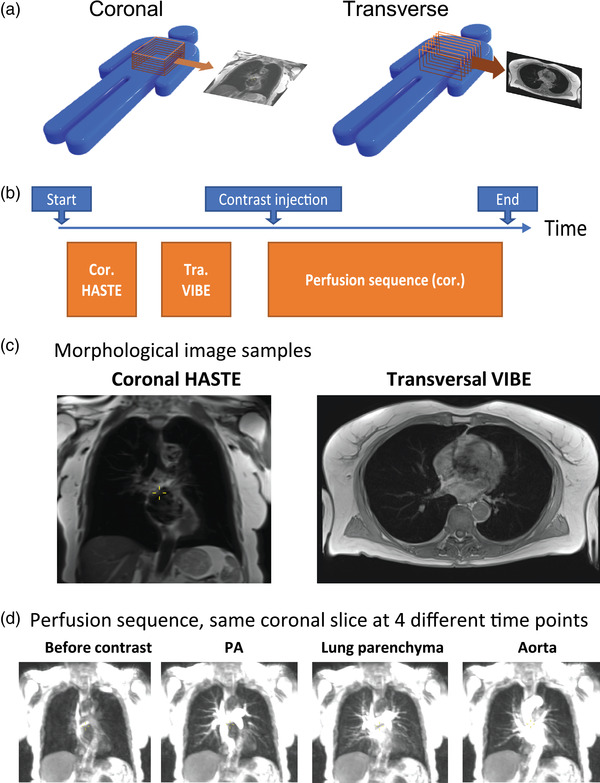
The imaging procedures and representative sample images of healthy controls and post‐COVID participants, respectively. (a) Illustration of the orientation of image stacks, with one sample slice on the side. (b) The imaging protocol and order of imaging sequences. (c) Each morphological sequence yields an image stack covering the thorax. The images show one sample slice from each morphological sequence (d). The perfusion sequence consists of a coronal image stack, repeated at an interval of 440 ms over 90 time points. The images below show one sample coronal slice at four key time points. Abbreviations: Cor, coronal; PA, pulmonary artery; Tra, transverse.

The DCE‐MRI was acquired through a 4D time‐resolved MRI angiography sequence with a keyhole T1‐weighted gradient‐recalled echo (“TWIST,” field of view 450 × 450 mm, voxel size 1.5 × 1.5 × 4 mm, echo/repetition times at 0.64/1.9 ms, 26 slices, a total of 90 phases in 40 s). A Spectris Solaris EP contrast injector (MEDRAD, Pittsburgh, USA; Bayer, Leverkusen, Germany) was used to administer gadoterate contrast agent (Clariscan, GE Healthcare, Chicago, USA), 0.5 mmol/ml, 2 ml, followed by 20 ml 0.9% saline solution at 5 ml/s. The patients were told to inhale and hold their breath for as long as possible during the 40‐s image acquisition, preceded by 10 deep breaths. The imaging procedures and representative example images are illustrated in Fig. 1. One DCE‐MRI time series is also included as Supplementary Information. Repeatability was evaluated by performing DCE‐MRI twice in five healthy controls.

### MRI image assessment and quantification

Morphological imaging was visually evaluated by J. Y. and independently verified by J. L. or S. N. (radiologists). MRI perfusion series were analyzed both visually and quantitatively on the entire imaging volume. Using Philips Intellispace (version 10.1.3, Philips Healthcare, Best, Netherlands), visual analysis was performed independently by J. Y. and S. N. using the method described by Eichinger et al. [33]. Quantitative analysis was performed using MATLAB (version R2020b, The Mathworks Inc., Natick, USA), using an in‐house developed post‐processing pipeline [18]. Briefly, both lungs were manually masked in the entire image volume of 26 slices, and regions of interest (ROI) were manually placed in the PA before its bifurcation and the aortic arch at its superior portion. ROIs were placed by J. Y. and independently verified by J. L. The TTP for contrast enhancement of each lung voxel and the ROIs were calculated using the MATLAB function max. To calculate the parametric TTP lung maps, each lung voxel TTP was normalized to the PA (as 0) and aorta (as 1) in the time domain. To allow for a structured and quantitative comparison between individuals and groups, the TTP maps were summarized into two scalar values: mean TTP and TTP ratio. Mean TTP was calculated as the average lung TTP values between PA and aorta. TTP ratio was calculated as the fraction of voxels with TTP values later than the aortic peak.

**Fig. 2:**
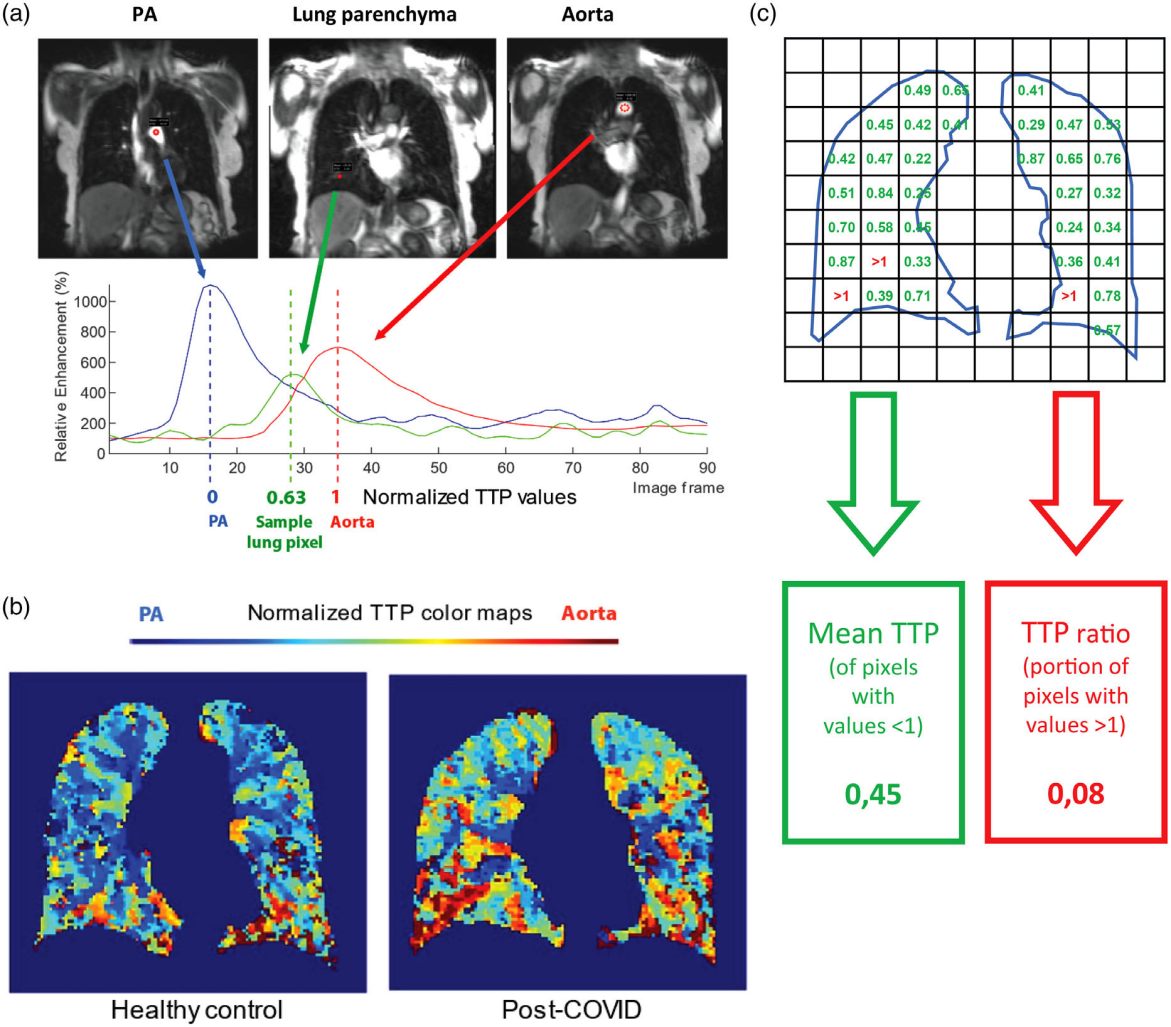
Post‐processing methodology. (a) Pictorial explanation of normalized time‐to‐peak (TTP) parametric map creation. Regions of interest (red circles) were placed in the pulmonary artery and aorta. Each mean enhancement curve was then calculated in the time domain (red and blue curves, respectively). For each lung pixel (only one is shown here), enhancement‐time data (the green curve) were used to find the corresponding TTP. The lung pixel TTP was then normalized to the timing of the TTP peaks in the pulmonary artery (0) and aorta (1). (b) Sample coronal slice of lung masked TTP map for healthy control (left) and post‐COVID participant (right). The colors represent normalized TTP values (PA/0 as blue, aorta/1 as red). (c) Calculation of summarizing parameters. Note: numbers and the exaggerated pixel size are for illustration purposes and do not mirror the actual data.

A graphic explanation of the perfusion quantification is shown in Fig. 2.

### Statistics analysis

All reported values are given as the mean and SD unless explicitly stated otherwise. Categorical variables were evaluated with the Pearson chi‐square test, and continuous variables were assessed with the Student's *t*‐test, while ordinal variables were evaluated with the Mann–Whitney test. When analyzing sex differences, either analysis of variance for continuous variables or Kruskal–Wallis for ordinal variables with Dunn–Sidak corrections for multiple comparisons was used since there is an uneven number of individuals to compare between multiple groups. Finally, correlations were assessed with the Spearman correlation method. Statistical analyses were performed using MATLAB (version R2020b, The Mathworks Inc., Natick, USA). To generate Figs 4 and 6, curves were smoothed using the moving mean method with a smoothing factor of 0.2, then interpolated using the modified Akima method. This was done to generate a unified X‐axis for simultaneous plotting. No interpolation was performed prior to calculating the mean TTP and TTP ratio.

## Results

**Fig. 3:**
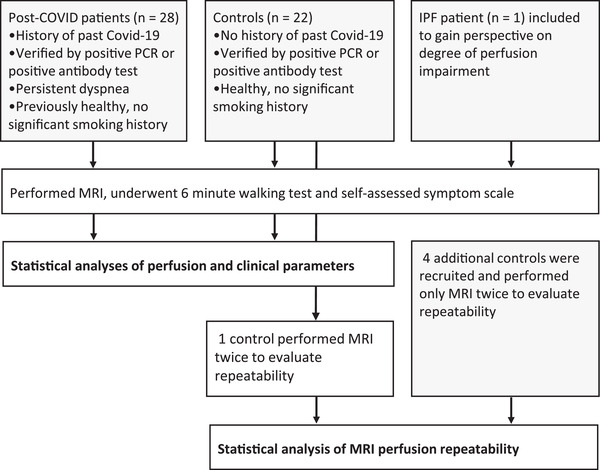
Flowchart of study participants. Abbreviations: IPF, idiopathic pulmonary fibrosis; MRI, magnetic resonance imaging; PCR, polymerase chain reaction.

In total, 51 participants were included: 28 individuals with longstanding symptoms and positive RT‐qPCR COVID‐19 test, 22 healthy controls, and one person with idiopathic pulmonary fibrosis. For brevity, the real‐time quantitative polymerase chain reaction (RT‐qPCR) positive group with longstanding symptoms following COVID‐19 will be referred to as the post‐COVID group. None in the post‐COVID group received treatment during the acute phase. All participants tolerated the scanning session well. Four additional persons were also included for repeatability testing of the DCE‐MRI setup. A flow chart of the study inclusions is presented in Fig. 3. The post‐COVID and healthy control groups did not differ with regard to age, gender distribution, weight, or BMI (Table 1). In the post‐COVID group, the mean time from symptom onset to the exam was 7.7 ± 3.6 months, with a biphasic distribution. About one‐half of the post‐COVID group performed imaging 4–6 months after symptom onset, and the rest 10–13 months after, reflecting the first and second wave of COVID‐19 pandemic in Sweden.

**Table 1:** All numbers are given as mean ± standard deviation, unless otherwise specified. Clinical and perfusion parameters comparison between the healthy and post‐COVID group

	Controls	Post‐COVID	Difference	*P*‐value
*N* (% females)	22 (73)	28 (75)		
Age (years)	44.1 (±10.8)	46.5 (±8)	2.4 (5%)	0.37^a^
Weight (kg)	73.8 (±12.1)	75.3 (±13.5)	1.5 (2%)	0.68^a^
Body mass index	25 (±3.4)	26 (±5.1)	1 (4%)	0.42^a^
6MWT (m)	678 (±78)	583 (±111)	−94 (−14%)	0.001^a^
6MWT (% of expected)	117 (±13)	102 (±18)	−15 (−13)	0.002^a^
Frändin–Grimby, current, median (IQR)	4 (2)	2.5 (1)	−1.5 (−38%)	<0.001^b^
Frändin–Grimby, previous, median (IQR)	4 (2)	5 (2)	1 (25%)	0.041^b^
MMRC, median (IQR)	1 (1)	2 (2)	−1 (100%)	<0.001^b^
CAT, median (IQR)	1 (2)	4 (1)	−3 (300%)	<0.001^b^
Mean TTP	0.41 (±0.03)	0.43 (±0.04)	0.03 (6%)	0.011^a^
TTP ratio	0.068 (±0.027)	0.096 (±0.052)	0.027 (40%)	0.032^a^

*Note*: CAT and mMRC are dyspnea scales; a high number means more dyspnea. Frändin–Grimby is a physical activity scale; a lower number means less physical activity. Mean TTP and TTP ratio are perfusion parameters; a higher number means more perfusion abnormality.

Abbreviations: CAT, Chronic Obstructive Pulmonary Disease Assessment Test; IQR, interquartile range; mMRC, modified Medical Research Council dyspnea scale; *N*, number of participants per group; TTP, time to peak; 6MWT, 6‐min walk test.

^a^ Student's *t*‐test.

^b^ Mann–Whitney U‐test.

The post‐COVID group had a lower self‐reported physical activity (2.5 ± 1, median, interquartile range [IQR]) than the control group (4 ± 2, median, IQR, *p* < 0.001) according to Frändin–Grimby. The recalled physical activity (5 ± 1, median, IQR) before COVID‐19 was higher than the controls (Table 1). Moreover, the post‐COVID group also reported more dyspnea on CAT (4 ± 1 vs. 1 ± 2, median, IQR, *p* < 0.001) and mMRC (2 ± 2 vs. 1 ± 1, median, IQR, *p* < 0.001), relative to the controls. There was no difference between men and women regarding physical activity or dyspnea (Table 2).

**Table 2:** All numbers are given as mean ± standard deviation (SD), unless otherwise specified; four‐way comparison of clinical and perfusion parameters

	Controls (females)	Controls (males)	Post‐COVID (females)	Post‐COVID (males)	Females, healthy vERSUs post‐COVID *p*‐values	Males, healthy vERSUs post‐COVID *p*‐values	Healthy females vERSUs males *p*‐values	Post‐COVID females vERSUs males *p*‐values
*N* (% OF GROUP)	16 (73)	6 (27)	21 (75)	7 (25)				
AGE, MEAN ± SD (YEARS)	44.5 (±10.1)	43 (±13.3)	46.5 (±7.4)	46.4 (±10.2)	0.99, ANOVA	0.99, ANOVA	1.000, ANOVA	1.000, ANOVA
WEIGHT, MEAN ± SD	71.3 (±11.7)	80.5 (±11.2)	73.3 (±12.8)	81.3 (±14.6)	1.0, ANOVA	1.00, ANOVA	0.58, ANOVA	0.63, ANOVA
BMI, MEAN ± SD (KG)	25.5 (±3.7)	23.8 (±1.8)	26.1 (±4.5)	25.7 (±6.9)	1.00, ANOVA	0.97, ANOVA	0.96, ANOVA	1.000, ANOVA
6MWT , MEAN ± SD (M)	666 (±81)	709 (±63)	561 (±104)	650 (±112)	0.008*, ANOVA	0.97, ANOVA	0.98, ANOVA	0.95, ANOVA
6MWT, MEAN ± SD (% EXPECTED)	118 (±13)	113 (±10)	101 (±18)	107 (±19)	0.009*, ANOVA	0.85, ANOVA	0.93, ANOVA	0.19, ANOVA
FRäNDIN–GRINBY CURRENT, MEDIAN (IQR)	4 (IQR 2)	4.5 (IQR 1)	3 (IQR 1)	2 (IQR 1.75)	0.001*, K‐W	0.002, K‐W	0.82, K‐W	1.00, K‐W
FRäNDIN–GRIMBY PREVIOUS, MEDIAN (IQR)	4 (IQR 2)	4.5 (IQR 1)	5 (IQR 1.5)	6 (IQR 1.75)	0.32, K‐W	0.73, K‐W	0.86, K‐W	0.65, K‐W
MMRC, MEDIAN (IQR)	1 (IQR 1)	0.5 (IQR 1)	2 (IQR 1.25)	2 (IQR 1.75)	<0.001*, K‐W	0.010*, K‐W	1.00, K‐W	1.00, K‐W
CAT, MEDIAN (IQR)	1 (IQR 1.5)	1 (IQR 1)	4 (IQR 1.25)	4 (IQR 1)	<0.001*, K‐W	<0.001*, K‐W	1.00, K‐W	0.99, K‐W
MEAN TTP, MEAN ± SD	0.41 (±0.03)	0.4 (±0.03)	0.42 (±0.04)	0.47 (±0.02)	0.87, ANOVA	0.001*, ANOVA	0.98, ANOVA	0.007*, ANOVA
TTP RATIO, MEAN ± SD	0.063 (±0.029)	0.082 (±0.027)	0.074 (±0.036)	0.16 (±0.039)	0.91, ANOVA	0.001*, ANOVA	0.84, ANOVA	<0.001*, ANOVA

*Note*: CAT and mMRC are dyspnea scales; a high number means more dyspnea. Frändin–Grimby is a physical activity scale; a lower number means less physical activity. Mean TTP and TTP ratio are perfusion parameters; a higher number means more perfusion abnormality.

Abbreviations: ANOVA, analysis of variance; CAT, Chronic Obstructive Pulmonary Disease Assessment Test; IQR, interquartile range; K‐W, Kruskal–Wallis; mMRC, modified Medical Research Council dyspnea scale; *N*, number of participants per group; TTP, time to peak; 6MWT, 6‐min walk test.

* *P* < 0.01.

When performing 6MWT, the post‐COVID group had a shorter walking distance, both in actual meters (583 ± 111 vs. 678 ± 78, *p* = 0.001) and normalized value, as a percentage of the expected reference value (102% ± 18% vs. 117% ± 13%, *p* = 0.002). However, both groups remained within the clinically normal range.

No systematic structural changes were identified on the morphological lung MRI imaging. Only small atelectatic opacities (maximum 7 mm) in 4/28 (14%) participants in the post‐COVID group were visualized. None of the controls had any detectable lung changes. In our visual assessment of the perfusion maps from the 4D time‐resolved MRI angiography sequence, we included an evaluation of large PA vessels. We did not detect any pulmonary embolisms. There was no correlation between time from symptom onset to MRI and any of the perfusion parameters (Table 3). However, we found the Frändin–Grimby score was lower by 3 ± 1.5 in the <8 months group compared to the >8 months group, 2 ± 1 (median, IQR, Mann–Whitney *p*‐value 0.02). There were no other systematic differences between the individuals who had their onset during the first/second wave, respectively (Table 3).

**Table 3:** All numbers are given as mean ± standard deviation, unless otherwise specified. Comparison within the post‐COVID group between those from the second wave and first wave, respectively

	<8 months	>8 months	*P*‐values
*N* (% females)	14 (71)	14 (79)	
Age (years)	44.7 (±9.2)	48.3 (±6.5)	0.245, STT
Weight (kg)	74.1 (±14.2)	76.5 (±13.2)	0.652, STT
BMI	25.7 (±5.2)	26.3 (±5.1)	0.751, STT
6MWT (m)	580 (±109)	587 (±117)	0.871, STT
6MWT (% of expected)	100 (±17)	104 (±19)	0.644, STT
Frändin–Grimby (current)	2 (±2)	3 (±2)	0.021, M‐W
Frändin–Grimby (before)	4.5 (±2)	5 (±2)	0.39, M‐W
mMRC	2 (±1)	2 (±1)	0.11, M‐W
CAT	5 (±1)	4 (±2)	0.12, M‐W
Months since acute infection	4.5 (±1)	11 (±1)	
Mean TTP	0.43 (±0.05)	0.440 (±0.030)	0.81, STT
TTP ratio	0.101 (±0.064)	0.090 (±0.040)	0.58, STT

Abbreviations: BMI, body mass index; CAT, Chronic Obstructive Pulmonary Disease Assessment Test; mMRC, modified Medical Research Council dyspnea scale; M‐W, Mann–Whitney U‐test; *N*, number of participants in the group; STT, Student's *t*‐test; TTP, time to peak; 6MWT, 6‐min walk test.

**Fig. 4:**
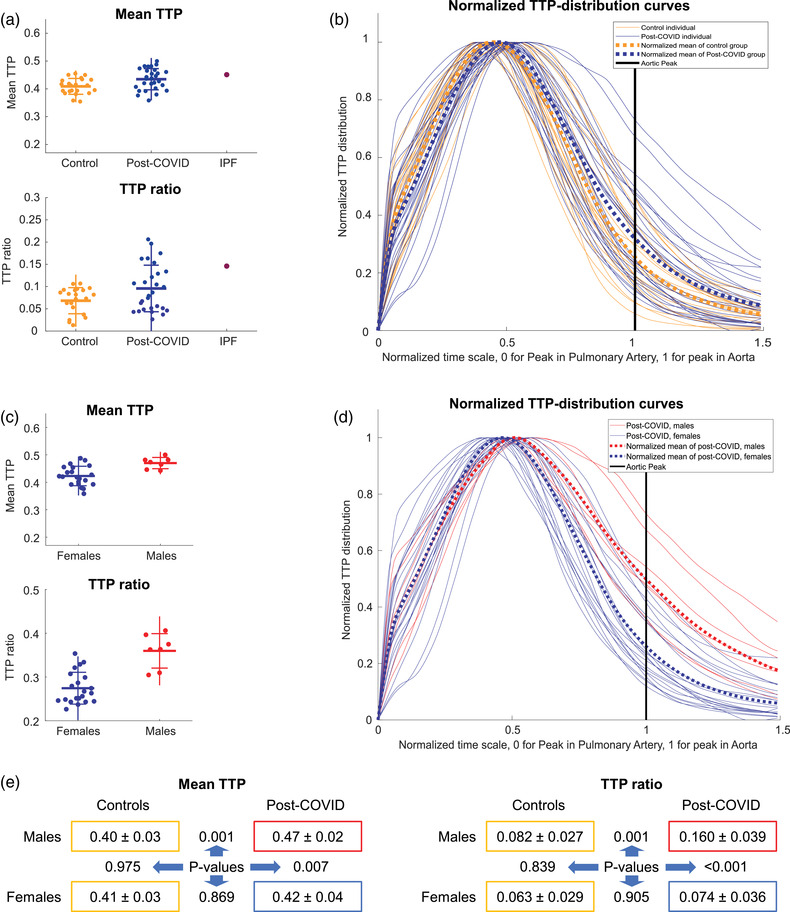
Perfusion results. (a) Mean time to peak (TTP) and TTP ratio scatterplot of individuals, grouped by healthy controls and post‐COVID participants. For reference, a person with light‐to‐moderate idiopathic pulmonary fibrosis (IPF) was added. (b) Individual TTP distribution curves comparing healthy and post‐COVID participants. Mean curve for each group in thick dotted lines. (c) Mean TTP and TTP ratio scatterplot of each individual from the post‐COVID group, grouped by sex. (d) Individual TTP distribution curves, comparing post‐COVID females and males. Mean curve for each group in thick dotted lines. (e) Numeric representation of perfusion parameters. Numbers following the “±” sign are standard deviations. The post‐COVID males and females are compared to each other and their healthy same‐sex controls using analysis of variance with Dunn–Sidak correction (p‐values between the colored boxes, significant values in red).

On the TTP parametric maps, there was visually a later contrast enhancement in many post‐COVID participants (Fig. 2). This is reflected in the mean TTP, being significantly higher in the post‐COVID group at 0.43 ± 0.04 versus 0.41 ± 0.03 (*p* = 0.011, Fig. 4). A higher mean TTP indicates an overall slower inflow of contrast bolus.

The TTP ratio, capturing bolus arrival later than the aortic peak in the lung parenchyma, was significantly higher in the post‐COVID group at 0.096 ± 0.052 versus 0.068 ± 0.027 in the control group (*p* = 0.032, Fig. 4). Visually, this is identified as focal areas of red on the TTP maps (Fig. 2). However, the concordance between the two readers was poor when doing a semiquantitative visual rating. The visual assessment results are described in detail in the Supplemental Information.

Notably, the post‐COVID group displayed a more considerable within‐group variability regarding TTP ratio and mean TTP (Fig. 4). The group differences are also reported in Table 2.

Subgroup analyses of a possible sex difference revealed that post‐COVID males had worse mean TTP (0.47 ± 0.02 vs. 0.40 ± 0.03, *p* = 0.001) and TTP ratio (0.160 ± 0.039 vs. 0.082 ± 0.027, *p* = 0.001) compared to the male control group. The comparisons are summarized in Fig. 4. The female post‐COVID group did not differ significantly from the female control group, neither regarding mean TTP nor TTP ratio. Notably, the female post‐COVID participants displayed greater variability in perfusion metrics relative to the female controls (Fig. 4).

**Fig. 5:**
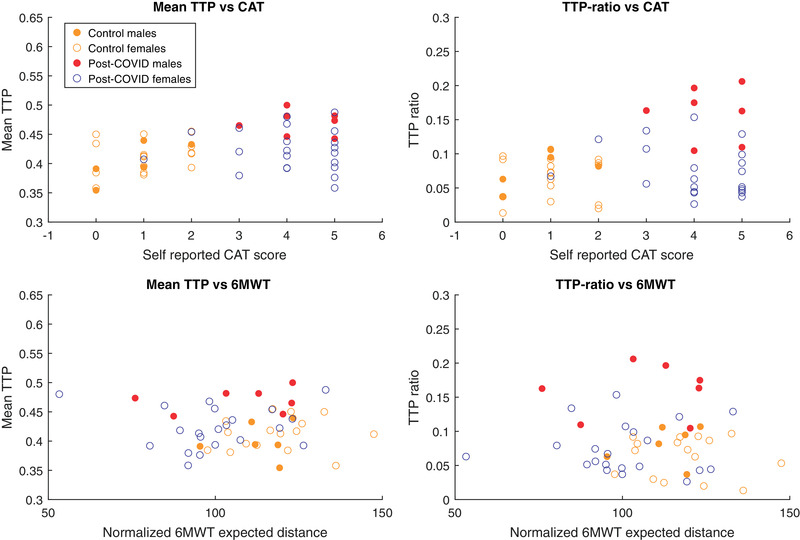
Perfusion—clinical correlation. Scatter plots for perfusion values and clinical assessments. Abbreviations: CAT, Chronic Obstructive Pulmonary Disease Assessment Test; TTP, time to peak; 6MWT, 6‐min walk test.

The overall correlation between self‐reported dyspnea, 6MWT, and TTP values are visualized as scatterplots (Fig. 5). When all participants were analyzed, we found a correlation between CAT and mean TTP (*r* = 0.35, *p* = 0.013). For males, this correlation was even more pronounced, both between CAT and mean TTP (*r* = 0.83, *p* = <0.001), and between CAT and TTP ratio (*r* = 0.76, *p* = 0.003). In contrast, no statistically significant correlation between CAT and any of the perfusion parameters was found in women. Results from 6MWT did not correlate to MRI‐DCE parameters.

To put the degree of perfusion impairment of the post‐COVID group into a clinical perspective, one male patient in his 80s with idiopathic pulmonary fibrosis was included (Fig. 4). His walking distance was 440 m (87% of the expected reference value). The TTP‐mean for this patient was 0.45 and the TTP‐ratio 0.15, which is comparable to the most pathological values seen in the post‐COVID group. This patient also had subtle peripheral morphologic changes involving a larger lung volume compared to the worst post‐COVID participants.

**Fig. 6:**
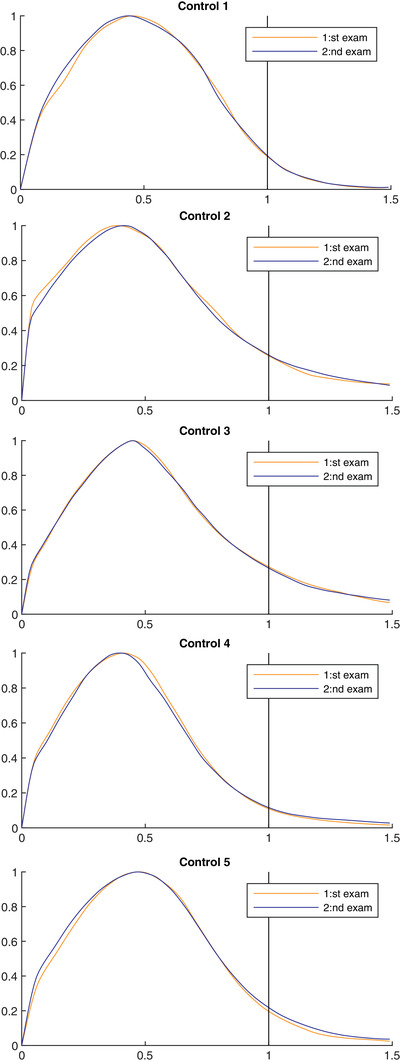
Repeatability control. Time‐to‐peak distribution curves from two separate exams in five healthy controls.

Five healthy controls performed DCE‐MRI twice with mean 14 ± 14 days in between, to evaluate the reproducibility. Over the two sessions, the difference in mean TTP was, on average, 0.016 ± 0.008 while TTP ratio varied, on average being 0.009 ± 0.006 (Fig. 6). The mean difference in mean TTP and TTP ratio between the males and females in the post‐COVID group was 0.047 and 0.085, respectively.

Diaphragm movement during the DCE sequence was also evaluated for all participants as a possible source of variability. The reference point for the movement was the superior aspect of the right diaphragm in the midcoronal slice (slice 13 of 26). The movement was quantified in the number of voxels during the bolus transit. Six participants had a diaphragm movement exceeding two voxels (3 mm), and the most considerable movement during bolus passage was six voxels (9 mm). Most (86%) of the participants could hold their breath during the entire 40‐s image acquisition. Seven subjects (14%) resumed breathing in the latter half, well after bolus transit.

## Discussion

Lung perfusion disturbances are a prominent pathophysiological feature during acute COVID‐19 and contribute to respiratory impairment [19]. Therefore, we wanted to examine lung perfusion in people with post‐COVID dyspnea to detect any abnormalities that may contribute to their symptoms. In order to do so, we chose a state‐of‐the‐art method, DCE‐MRI, suitable for young patients and repeated imaging sessions. We produced TTP maps normalized in the time domain from the perfusion images, which show how fast the lung parenchyma enhances with contrast. To compare the groups with greater objectivity and precision, we summarized these quantitatively into two perfusion parameters: mean TTP and TTP ratio. We consider mean TTP to represent an overall speed of enhancement, in which a higher value would represent a higher perfusion resistance. TTP‐ratio is the portion of the lung that has contrast enhancement later than the aortic peak. Notably, we detected high mean TTP and high TTP ratio in males from a prospective convenience sample of nonhospitalized post‐COVID patients. In addition, there was a significant correlation with dyspnea in males but not in females. Consequently, our findings could be a step forward in explaining post‐COVID dyspnea, of which the mechanisms are still largely unknown, especially in nonhospitalized patients. However, the lack of perfusion disturbances in women needs attention.

Sex differences in pathophysiology have been reported regarding acute COVID‐19. Men have a higher expression of angiotensin‐converting enzyme II receptor, abundant in the lung, and a higher propensity for COVID‐associated respiratory failure and mortality [34]. Thus, the lack of perfusion disturbances in women could perhaps be anticipated. We should point out that our results do not exclude the possibility of lung perfusion disturbances in females. We observed considerable variability in the female group, with some individuals showing impaired perfusion comparable to the males. Several possibilities for this discrepancy can be considered here, including sex differences in self‐reported dyspnea, peripheral airway obstruction, weakness in respiratory muscles, and perhaps perfusion disturbances in peripheral muscles [35, 36].

An essential factor to discuss is that the imaging was conducted during rest and not exertion. Pulmonary perfusion is directly linked to cardiac output, which can increase manyfold during heavy physical exertion. In addition, lung perfusion becomes more heterogeneous as pulmonary blood flow increases [37]. This increase might be more pronounced in individuals with post‐COVID‐19 condition. Due to practical reasons, it is challenging to perform DCE‐MRI during physical exertion, which applies to other perfusion imaging methods as well. A possible extension of the current work could be pharmacologically induced stress testing during DCE‐MRI, thereby attenuating possible group differences.

Based on our results, we believe that this lung DCE‐MRI method is feasible for exploring lung perfusion in a larger clinical setting with the advantage of it being extendable to any MRI scanner. While our exam would not reveal the exact cause of perfusion disturbances, when done in conjunction with other diagnostic and interventional trials, the underlying cause of perfusion disturbances may be further elucidated, be it microthrombi, parenchymal destruction, dysregulation, or possibly secondary to ventilation disturbances. DCE‐MRI was chosen as our study method for its many advantages, including temporal information and lack of radiation. Its application in lung perfusion imaging in other conditions has thus far yielded convincing results [23]. Analysis of TTP parametric maps in high‐altitude pulmonary edema susceptible individuals has previously been reported [38]. The post‐processing method whereby the TTP maps are normalized in the time domain and summarized into mean TTP and TTP ratio is straightforward and has been successfully applied in one patient treated with intensive care for COVID‐19 and a porcine model simulating severe COVID‐19 [18]. The repeatability of lung perfusion MRI has previously been shown to be reasonably good [39]. Our repeatability assessments of five healthy participants have produced an average normalized mean TTP variability of 0.015 ± 0.008 and an average TTP ratio variability of 0.009 ± 0.006, below the group difference between the male post‐COVID participants and other groups.

To our knowledge, this is the largest cohort examined using lung DCE‐MRI in post‐COVID. These patients did, by definition, not require hospitalization or any significant medical treatment during their acute phase, possibly representing a “mild” stratum of patients. Our findings thus indicate a possible physiological disturbance beyond morphological changes, similar to other studies using DECT or hyperpolarized ^129^Xe MRI [6, 40]. What is surprising is our finding of perfusion disturbances up to 1 year after symptom onset. This finding could suggest a long‐lasting or even perpetual lung injury in some patients. Further studies would elucidate the factors predicting long‐term outcomes.

This study has some limitations, including a lack of other supporting imaging modalities. CT scans would likely detect morphological lung changes with higher sensitivity, but the current MRI protocol was optimized with advanced morphological imaging, including ultrashort echo time imaging that has recently been successfully applied in cystic fibrosis [41]. This study can be regarded as exploratory on the potential use of DCE‐MRI in post‐COVID, and future studies should combine multiple imaging modalities such as CT, echocardiography, and pulmonary physiological measurements such as spirometry. We did not perform a proper high‐resolution MR angiography, as this would require a significantly higher gadolinium contrast dosage (0.2 mmol/kg, up to 20 ml). In addition, given their normal 6MWT results, we had virtually no clinical suspicion anyone would have a large pulmonary embolism and were thus not inclined to perform MR angiography. Further, the DCE‐MRI sequence visualizes central arteries out to the segment arteries, and no emboli could be detected. A technical limitation that is hard to mitigate is the possible contribution of inspiration during the expected breath‐hold. A higher degree of inspiration can increase pulmonary resistance by stretching alveolar capillaries and by vessel compression due to higher intrathoracic pressure [42]. Nevertheless, the repeatability of five healthy volunteers was excellent. In addition, the unstructured recruitment of patients through a network of self‐identified “long haulers” following COVID‐19 could be considered both a weakness and a strength. The potentially increased heterogenicity is offset by a better reflection of clinical reality. In hindsight, with regard to perfusion disturbances detected predominantly in men, the male participants would ideally have been more numerous. It could be argued that the clinical tests we used, originally meant for other debilitating lung diseases, were not an optimal match for our participants. In addition, post hoc assessments of physical activity before COVID‐19 may also be subject to a recall bias. Nevertheless, our results suggest that perfusion impairment contributes to dyspnea and reduced exercise capacity in our sample of nonhospitalized post‐COVID individuals.

In conclusion, by calculating the quantitative parameters from MRI images, lung perfusion disturbances can be detected in nonhospitalized males long after an acute COVID‐19 infection. However, there were no significant perfusion disturbances in females despite an equal level of dyspnea. This finding implies a sex difference in post‐COVID dyspnea pathophysiology, which needs to be considered in future studies. While this exam cannot alone differentiate the cause of perfusion disturbances, be it primary vascular lesions or secondary to hypoxic vasoconstriction, we believe that DCE‐MRI could ideally be combined with other functional imaging modalities to elucidate the post‐COVID pathophysiology further.

## Funding

This study was supported by grants from the Swedish Heart and Lung Foundation (No. 20210114) and Karolinska Institutet. J. L. was supported by MedTechLabs and a private donation by Tedde Jeansson Sr.

## Conflict of interest

The authors declare no conflict of interest.

## Author contributions

Jimmy Z. Yu: Conceptualization; Data curation; Formal analysis; Investigation; Methodology; Software; Visualization; Writing‐original draft; Writing‐review and editing. Tobias Granberg: Conceptualization; Data curation; Funding acquisition; Investigation; Methodology; Resources; Supervision; Writing‐review and editing. Roya Shams: Investigation; Writing‐review and editing. Sven Petersson: Investigation; Methodology; Writing‐review and editing. Sven Nyrén: Formal analysis; Methodology; Supervision. Johan Lundberg: Conceptualization; Data curation; Formal analysis; Methodology; Software; Supervision; Visualization; Writing‐review and editing.

## Supporting information


**Supplementary Figure 1**: Scatter plots showing associations between mean time‐to‐peak (TTP) and TTP ratio compared to Age, Body mass Index (BMI), 6‐minute walking test (6MWT), modified Medical Research Council dyspnea scale (mMRC), Chronic Obstructive Pulmonary Disease assessment test (CAT), daily activity, and symptom duration.
**Supplementary Table 1**: Contingency table of results from both readers.
**Supplementary Table 2**: Comparison of visual grading on the lobar level and on the total score.

## Data Availability

The anonymized datasets generated and analyzed in the study are available from the corresponding author on reasonable request.
