# Supplementary material for: Lung perfusion disturbances in nonhospitalized post‐COVID with dyspnea—A magnetic resonance imaging feasibility study
Source: J Intern Med. 2022 Aug 26:10.1111/joim.13558. Online ahead of print. doi: 10.1111/joim.13558 (PMC9539011; doi:10.1111/joim.13558)
Supplement: Supplementary file 1 — Supplementary Figure 1: Scatter plots showing associations between mean time‐to‐peak (TTP) and TTP ratio compared to Age, Body mass Index (BMI), 6‐minute walking test (6MWT), modified Medical Research Council dyspnea scale (mMRC), Chronic Obstructive Pulmonary Disease assessment test (CAT), daily activity, and symptom duration. Supplementary Table 1: Contingency table of results from both readers. Supplementary Table 2: Comparison of visual grading on the lobar level and on the total score. [file JOIM-9999-0-s002.pdf]

Lung perfusion disturbances in non-hospitalized post-COVID with dyspnea – An MRI feasibility study

Supplementary Information

Jimmy Yu,[1,2] Tobias Granberg,[3,4] Roya Shams,[3] Sven Petersson,[4,5] Magnus Sköld,[6, 7] Sven Nyrén,[1,2] Johan Lundberg,[3,4]

1. **Department of Radiology Solna, Karolinska University Hospital, Stockholm, Sweden.**
2. **Department of Molecular Medicine and Surgery, Karolinska Institutet, Stockholm, Sweden.**
3. **Department of Neuroradiology, Karolinska University Hospital, Stockholm, Sweden.**
4. **Department of Clinical Neuroscience, Karolinska Institutet, Stockholm, Sweden.**
5. **Department of Medical Radiation Physics and Nuclear Medicine, Karolinska University Hospital, Stockholm, Sweden.**
6. **Department of Respiratory Medicine and Allergy, Karolinska University Hospital, Stockholm, Sweden.**
7. **Department of Medicine Solna, Karolinska Institutet, Stockholm, Sweden.**

Supplementary Methods

Visual assessment

We performed a structured visual assessment of perfusion defects by generating color and greyscale parametric maps of pulmonary blood volume (PBV), pulmonary blood flow (PBF), and time-to-peak (TTP) from the entire image stack (26 slices) using Philips Intellispace (version 10.1.3, Philips Healthcare, Best, Netherlands). The entire parametric map stacks were visually assessed for focal areas of low PBV, PBF, and late TTP, according to the appearances described by Risse et al. [1]. Due to the fractal structure of lung vessels and therefore heterogeneous distribution of blood flow, as defined by Glenny et al. [2] the maps were accordingly heterogeneous in appearance. Subsequently, for a perfusion defect to be detected visually, it must be large enough to transcend the background anatomical heterogeneity. Judging from images of the sample perfusion defects described by Risse et al. [1] the perfusion defects need to be at least segmental in size to detect visually. Unfortunately, deficiencies of this size couldn't readily be identified in our images, perhaps since perfusion defects during COVID-19 can have a "mottled" appearance [3].

What could be more readily identified was that some participants in the post-COVID group tend to have generally higher values in the time-domain normalized TTP maps, representing later contrast arrival. Nevertheless, we did discern some smaller-than-subsegmental peripheral areas of suspected perfusion defects and thus attempted systematic visual analysis using the protocol from Eichinger et al. [4]. Briefly, each lobe was assessed and assigned a semi-quantitative score, ranging from 0–2 (0 = no abnormality, 1 = <50% of the lobe involved, 2 = >50% of the lobe involved). Finally, the sum of scores for each lobe was summed as a total score ranging from 0 to 12.

Initially, the comparison was made dichotomously on an individual level by classifying the total score of 0 as "no perfusion defects" and anything above as "perfusion defects detected". Next, the concordance between the two readers was analyzed using Cohen's kappa, resulting in an unweighted kappa value of -0.02, equaling "poor" agreement. The results are presented in the tables below.

Supplementary Table 1

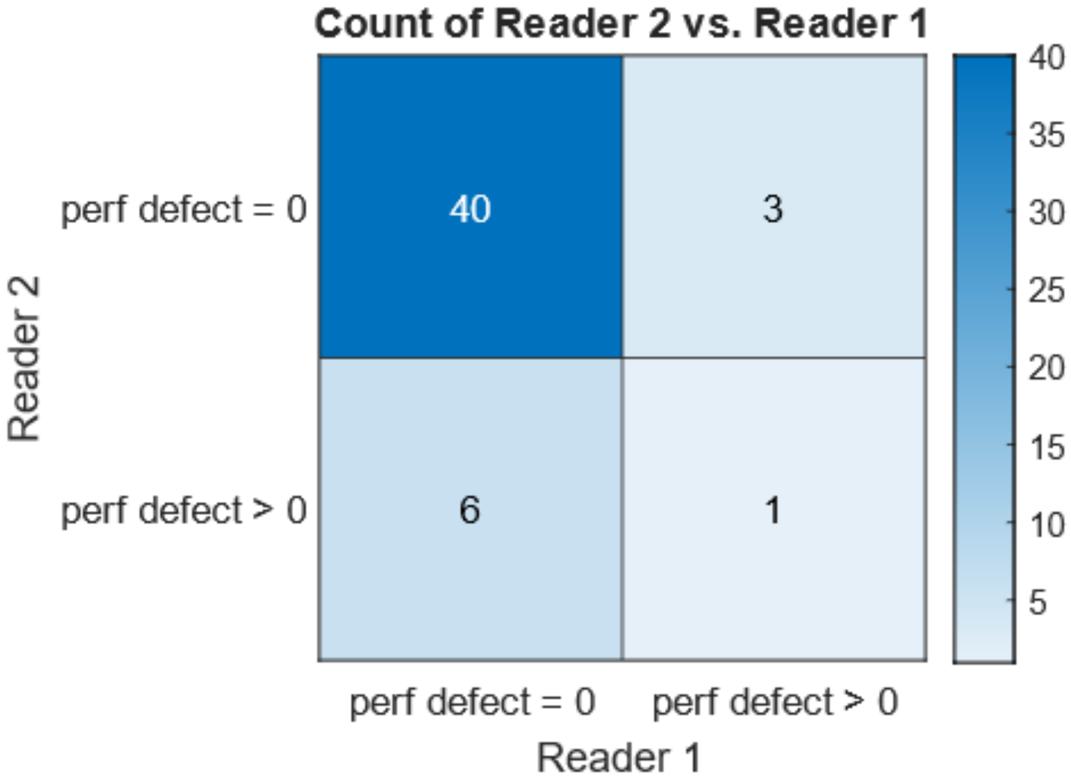

Contingency table of results from both readers.

Supplementary Table 2

Parti-cipant	Rater 1 score							Rater 2 score							Rater 1 vs rater 2 difference						
	RUL	RML	RLL	LUL	LML	LLL	Sum	RUL	RML	RLL	LUL	LML	LLL	Sum	RUL	RML	RLL	LUL	LML	LLL	Sum
1	0	0	0	0	0	0	0	0	0	0	0	0	1	1	0	0	0	0	0	-1	-1
2	0	0	0	0	0	0	0	0	1	0	0	0	0	1	0	-1	0	0	0	0	-1
3	0	0	0	0	0	0	0	0	0	0	0	0	0	0	0	0	0	0	0	0	0
4	0	0	0	0	0	0	0	0	0	0	0	0	0	0	0	0	0	0	0	0	0
5	0	0	0	0	0	0	0	0	0	0	0	0	0	0	0	0	0	0	0	0	0
6	0	0	1	0	0	1	2	1	0	0	1	0	0	2	-1	1	0	-1	1	2	0
7	0	0	0	0	0	1	1	0	0	0	0	0	0	0	0	0	0	0	1	1	1
8	0	0	0	0	0	0	0	0	0	0	0	0	0	0	0	0	0	0	0	0	0
9	0	0	0	0	0	0	0	0	0	1	0	1	0	2	0	0	-1	0	-1	0	-2
10	0	0	1	0	0	1	2	0	0	0	0	0	0	0	0	1	0	0	1	2	2
11	0	0	0	0	0	0	0	0	0	0	0	0	0	0	0	0	0	0	0	0	0
12	0	0	0	0	0	0	0	0	0	0	0	0	0	0	0	0	0	0	0	0	0
13	0	0	0	0	0	0	0	0	0	0	0	0	0	0	0	0	0	0	0	0	0
14	0	0	0	0	0	0	0	0	0	0	0	0	0	0	0	0	0	0	0	0	0
15	0	0	0	0	0	0	0	0	0	0	0	0	0	0	0	0	0	0	0	0	0
16	0	0	0	0	0	0	0	0	0	0	0	0	0	0	0	0	0	0	0	0	0
17	0	0	0	0	0	0	0	0	0	0	0	0	0	0	0	0	0	0	0	0	0
18	1	0	0	0	0	0	1	0	0	0	0	0	0	0	0	0	0	0	0	1	1
19	0	0	0	0	0	1	1	0	0	0	0	0	0	0	0	0	0	0	1	1	1
20	0	0	0	0	0	0	0	0	0	0	0	0	0	0	0	0	0	0	0	0	0
21	0	0	0	0	0	0	0	0	0	0	0	0	0	0	0	0	0	0	0	0	0
22	0	0	0	0	0	1	1	0	0	0	0	0	0	0	0	0	0	0	1	1	1
23	0	0	0	0	0	0	0	0	0	0	0	0	0	0	0	0	0	0	0	0	0
24	0	0	0	0	0	0	0	0	0	0	0	0	0	0	0	0	0	0	0	0	0
25	0	0	0	0	0	1	1	0	0	0	0	0	0	0	0	0	0	0	1	1	1
26	0	0	0	0	0	0	0	0	0	0	0	0	0	0	0	0	0	0	0	0	0
27	0	0	0	0	0	0	0	0	0	0	0	0	0	0	0	0	0	0	0	0	0
28	0	0	0	0	0	0	0	0	0	0	0	0	0	0	0	0	0	0	0	0	0
29	0	0	1	0	0	1	2	0	0	0	0	0	0	0	0	1	0	0	1	2	2
30	0	0	0	0	0	0	0	0	0	0	0	0	0	0	0	0	0	0	0	0	0
31	0	0	0	0	0	0	0	0	0	0	0	0	0	0	0	0	0	0	0	0	0
32	0	0	0	0	0	0	0	0	0	0	0	0	0	0	0	0	0	0	0	0	0
33	0	0	0	0	0	0	0	2	1	0	2	0	0	5	-2	-1	0	-2	0	0	-5
34	0	0	0	0	0	0	0	0	0	0	0	0	0	0	0	0	0	0	0	0	0
35	0	0	0	0	0	1	1	0	0	1	0	0	0	1	0	0	-1	0	1	1	0
36	0	0	0	0	0	0	0	0	0	0	0	0	0	0	0	0	0	0	0	0	0
37	0	0	0	0	0	0	0	0	0	0	0	0	0	0	0	0	0	0	0	0	0
38	0	0	0	0	0	0	0	1	0	0	1	0	0	2	-1	0	0	-1	0	0	-2
39	0	0	0	0	0	0	0	0	0	0	0	0	0	0	0	0	0	0	0	0	0
40	0	0	0	0	0	0	0	0	0	0	0	0	0	0	0	0	0	0	0	0	0
41	0	0	0	0	0	0	0	0	0	0	0	0	0	0	0	0	0	0	0	0	0
42	0	0	0	0	0	1	1	0	0	0	0	0	0	0	0	0	0	0	1	1	1
43	0	0	0	0	0	0	0	0	0	0	0	0	0	0	0	0	0	0	0	0	0
44	0	0	0	0	0	0	0	0	0	0	0	0	0	0	0	0	0	0	0	0	0
45	0	0	0	0	0	0	0	0	0	0	0	0	0	0	0	0	0	0	0	0	0
46	0	0	0	0	0	0	0	0	0	0	0	0	0	0	0	0	0	0	0	0	0
47	0	0	0	0	0	0	0	0	0	0	0	0	0	0	0	0	0	0	0	0	0
48	0	0	0	0	0	0	0	0	0	0	0	0	0	0	0	0	0	0	0	0	0
49	0	0	0	0	0	0	0	0	0	0	0	0	0	0	0	0	0	0	0	0	0
50	0	0	0	0	0	0	0	0	0	0	0	0	0	0	0	0	0	0	0	0	0

Comparison of visual grading on the lobar level and on the total score. Abbreviations: RUL – right upper lobe, RML – right middle lobe, RLL – right lower lobe, LUL – left upper lobe, LML – left middle lobe (lingula), LLL – left lower lobe.

Supplementary Figure 1

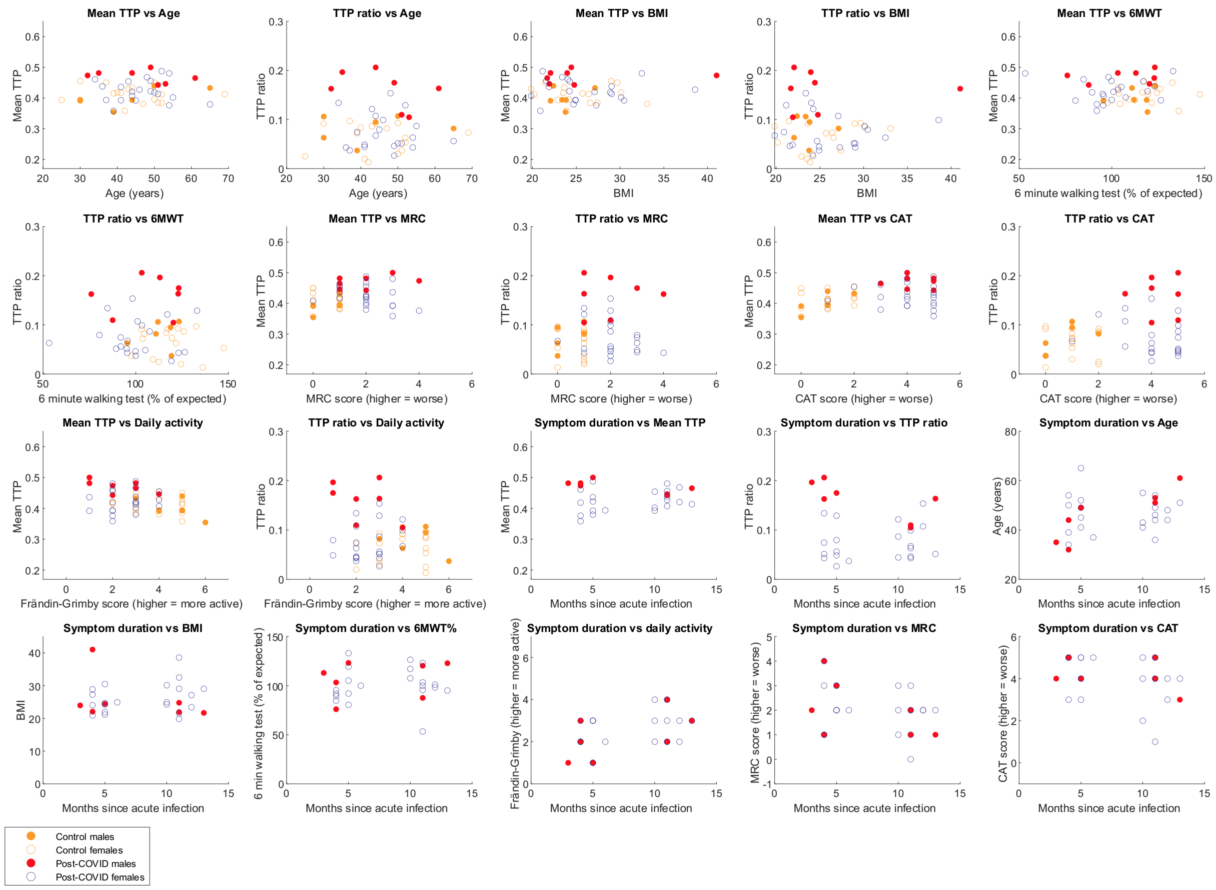

Scatter plots showing associations between mean time-to-peak (TTP) and TTP ratio compared to Age, Body mass Index (BMI), 6-minute walking test (6MWT), modified Medical Research Council dyspnea scale (mMRC), Chronic Obstructive Pulmonary Disease assessment test (CAT), daily activity, and symptom duration.

Supplementary References

1. Risse F, Eichinger M, Kauczor HU, Semmler W, Puderbach M. Improved visualization of delayed perfusion in lung MRI. *European journal of radiology* 2011; 77: 105–10.

2. Glenny RW, Bernard SL, Robertson HT. Pulmonary blood flow remains fractal down to the level of gas exchange. *J Appl Physiol* 2000; 89: 742–8.

3. Dhawan RT, Gopalan D, Howard L, *et al.* Beyond the clot: perfusion imaging of the pulmonary vasculature after COVID-19. *Lancet Respir Medicine* 2020; 9: 107–16.

4. Eichinger M, Optazaite D-E, Kopp-Schneider A, *et al.* Morphologic and functional scoring of cystic fibrosis lung disease using MRI. *Eur J Radiol* 2012; 81: 1321–9.
